# Expression of Concern: The protective effect of PPARγ in sepsis-induced acute lung injury via inhibiting PTEN/β-catenin pathway

**DOI:** 10.1042/BSR-20192639_EOC

**Published:** 2020-06-24

**Keywords:** sepsis, acute lung injury, PPARγ, PTEN/β-catenin pathway, apoptosis, inflammation

The Editorial Office has been made aware of potential issues surrounding the scientific validity of this paper, hence has issued an expression of concern to notify readers whilst the Editorial Office investigates.

